# Molecular Mechanisms of Lipoic Acid Protection against Aflatoxin B_1_-Induced Liver Oxidative Damage and Inflammatory Responses in Broilers

**DOI:** 10.3390/toxins7124879

**Published:** 2015-12-14

**Authors:** Qiugang Ma, Yan Li, Yu Fan, Lihong Zhao, Hua Wei, Cheng Ji, Jianyun Zhang

**Affiliations:** 1 State Key Laboratory of Animal Nutrition, College of Animal Science and Technology, China Agricultural University, Beijing 100193, China; maqiugang@cau.edu.cn (Q.M.); liyan-602@163.com (Y.L.); fanyucau@163.com (Y.F.); lihongzhao100@126.com (L.Z.); jicheng@cau.edu.cn (C.J.); 2 Translational Medicine Lab, Chinese National Human Genome Center, Beijing 100176, China; huaweimda@126.com

**Keywords:** lipoic acid, aflatoxin B_1_, liver, NF-κB, inflammation, oxidative stress

## Abstract

Alpha-lipoic acid (α-LA) was evaluated in this study for its molecular mechanisms against liver oxidative damage and inflammatory responses induced by aflatoxin B_1_ (AFB_1_). Birds were randomly allocated into four groups with different diets for three weeks: a basal diet, a 300 mg/kg α-LA supplementation in a basal diet, a diet containing 74 μg/kg AFB_1_, and 300 mg/kg α-LA supplementation in a diet containing 74 μg/kg AFB_1_. In the AFB_1_ group, the expression of *GSH-P_X_* mRNA was down-regulated (*p* < 0.05), and the levels of lipid peroxide and nitric oxide were increased (*p* < 0.05) in the chicken livers compared to those of the control group. Additionally, the mRNA level of the pro-inflammatory factor interleukin-6 was up-regulated significantly (*p* < 0.05), the protein expressions of both the nuclear factor kappa B (NF-κB) p65 and the inducible nitric oxide synthase were enhanced significantly (*p* < 0.05) in the AFB_1_ group. All of these negative effects were inhibited by α-LA. These results indicate that α-LA may be effective in preventing hepatic oxidative stress, down-regulating the expression of hepatic pro-inflammatory cytokines, as well as inhibiting NF-κB expression.

## 1. Introduction

Mycotoxins are a group of secondary fungal metabolites that occur widely in natural contaminants of many feeds and foods under conditions of high humidity and temperature, and are potentially hazardous to animal and human health. Approximately 25% of the world’s grain production is contaminated with mycotoxins, indicating the global prevalence of this problem. Aflatoxin B_1_ (AFB_1_), a naturally occurring mycotoxin, is a potent teratogenic, mutagenic, immunotoxic, hepatotoxic, and nephrotoxic agent [1]. AFB_1_ significantly constrains the development of animal husbandry and poses a health risk to people, even leading to hepatocellular carcinoma due to its mutagenic effect. In addition, aflatoxin-contaminated animal products (such as animal tissues, milk, and eggs) can lead to the transfer of toxins through the food chain and affect human health. Therefore, AFB_1_ has raised concerns globally due to its substantial risk to the market economy as well as human public health.

Previous studies have documented that AFB_1_ reduces growth performance and immune function, induces oxidative stress, alters blood profiles and gut morphology, and partially damages internal organs in animals [2,3]. The varied effects of AFB_1_ toxicity *in vivo* depend on the dosage and duration of exposure, age, and sex of the exposed individuals, genetics, health and nutritional status, and animal species. In the current study, low dosages of AFB_1_ were chosen mainly because the occurrence and contamination levels of AFB_1_ under natural conditions have commonly been found to be relatively low. In addition, long term consumption of low concentrations of AFB_1_ can be harmful to animal and human health, due to effects such as markedly impairing liver function, the immune system, and oxidative defense mechanisms [4]. Oxidative stress plays a major role in aflatoxicosis. It has been reported that AFB_1_ can induce the production of free radicals and lipid peroxides, resulting in cell damage [5]. Therefore, some antioxidants might be useful in preventing or attenuating the detrimental effects of chronic AFB_1_ toxicity in animals.

Alpha-lipoic acid (α-LA) is an orthomolecular nutrient found in broccoli, collards, spinach, beef, and organ meats (which contain small amounts of LA). It is well known as an “ideal antioxidant”, possessing many beneficial properties, including the ability to chelate metals, quench specific radicals, and regenerate other antioxidants such as ascorbate, vitamin E, and glutathione (GSH) [6], along with regulating the activity of transcription factors such as the nuclear factor kappa B (NF-κB) [7]. Therefore, it has been used for treating diseases in which oxidative stress plays a critical role [6]. Karaman *et al.* [8] reported that the supplementation of antioxidants (LA) protected the liver from lipid peroxidation caused by aflatoxin (AF) (150 and 300 ppb). Our previous studies have also shown that the supplementation of α-LA into AFs free-diets could enhance the antioxidant capability of broilers [9,10] and piglets [11].

The liver is the main organ of detoxification and oxidation in the body, exhibiting a high metabolic rate. It is also considered as an immunological organ [12]. It has been demonstrated that α-LA has potent antioxidant and anti-inflammatory properties [13], which might play an important role in protecting the liver from damage caused by AFB_1_. However, the protective molecular mechanism of α-LA against liver injury caused by AF *in vivo* has not been completely revealed. Therefore, the present study evaluates and further explores these underlying mechanisms. We hypothesized that α-LA may protect the liver from AFB_1_-induced damage by attenuating oxidative stress, suppressing the inflammatory response, and inhibiting the NF-κB expression. As NF-κB (a redox-sensitive transcription factor) is known to play an important role in modulating the expression of a variety of cellular genes that participate in cytokine production, inflammation, and apoptosis [14], we also investigated the possibility that NF-κB might be a possible target of α-LA-mediated protection from AFB_1_-induced hepatoxity. To our knowledge, this is the first study to explore the role of NF-κB signaling in the protective effects of α-LA against AFB_1_-induced liver damage.

## 2. Results

### 2.1. Effects of α-LA on the mRNA Levels of Antioxidant Genes in Livers of Chickens Exposed to AFB_1_

**Figure 1:**
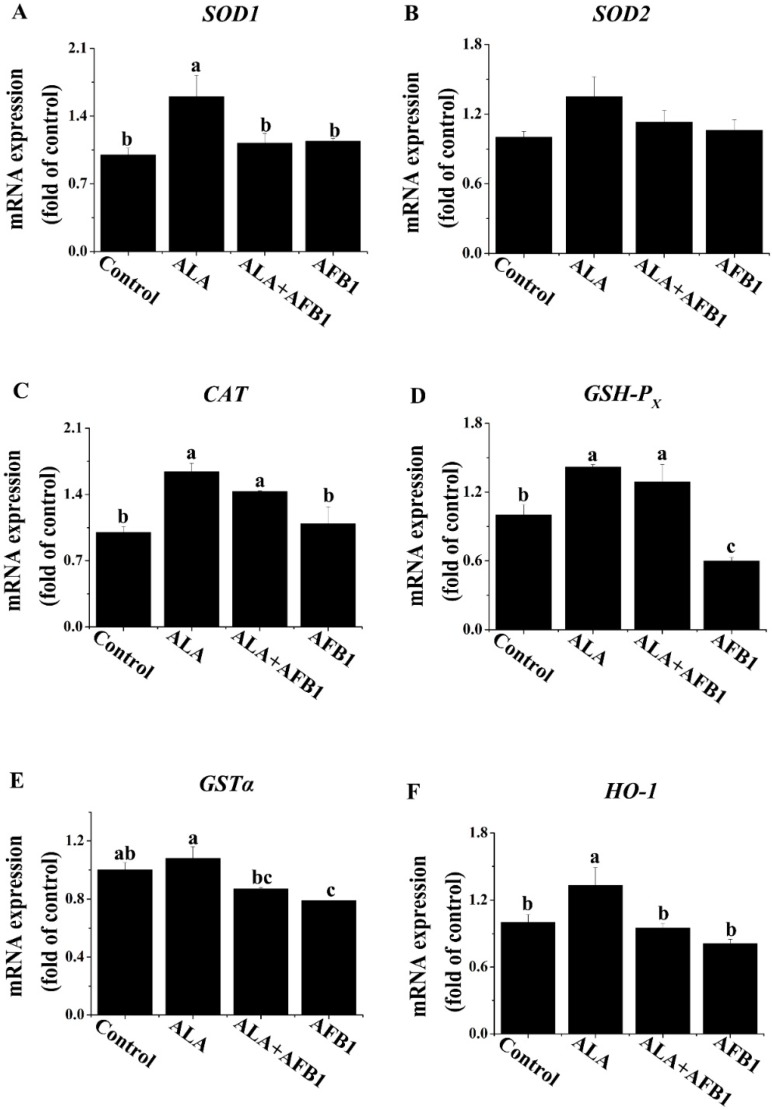
Effect of lipoic acid on the expression of antioxidant genes of the liver in broilers fed a diet containing aflatoxin B_1_ (AFB_1_). Values are means ± SE (*n* = 8 per group). Columns with different letters are significantly different (*p* < 0.05). (**A**) *SOD1*, Cu/Zn superoxide dismutase; (**B**) *SOD2*, Mn superoxide dismutase; (**C**) *CAT*, catalase activity; (**D**) *GSH-P_X_*, glutathione peroxidase; (**E**) *GSTα*, glutathione S transferase alpha; (**F**) *HO-1*, heme oxygenase-1; SE, standard error.

Changes in antioxidant genes are generally acknowledged by the actions of transcriptional regulators. The expression levels of Cu/Zn superoxide dismutase (*SOD1*), Mn superoxide dismutase (*SOD2*), catalase activity (*CAT*), glutathione peroxidase (*GSH-P_X_*), glutathione S transferase alpha (*GSTα*), and heme oxygenase-1 (*HO-1*) mRNA in the livers of all four groups were evaluated by RT qPCR (Figure 1). *GSH-P_X_* and *GSTα* mRNA expression levels were decreased greatly (*p* < 0.05; Figure 1D,E) in the livers of birds exposed to AFB_1_ as compared with the birds from the control group. The decreased *GSH-P_X_* expression in the livers of birds under AFB_1_ treatment was normalized completely by the treatment with α-LA (Figure 1D). Additionally, the *GSTα* mRNA expression in the AFB_1_ plus α-LA group was similar to that in control group (Figure 1E). Treatment with an AFB_1_ plus α-LA diet slightly enhanced *CAT* mRNA expression as compared to birds fed the diet containing AFB_1_ only. Moreover, compared to the control diet, α-LA alone in the diet increased (*p* < 0.05) the hepatic *SOD1*, *CAT*, *GSH-P_X_*, and *HO-1* gene expression (Figure 1A,C,D,F).

### 2.2. Effects of α-LA on the Levels of LPO and NO in Livers of Chickens Exposed to AFB_1_

**Figure 2:**
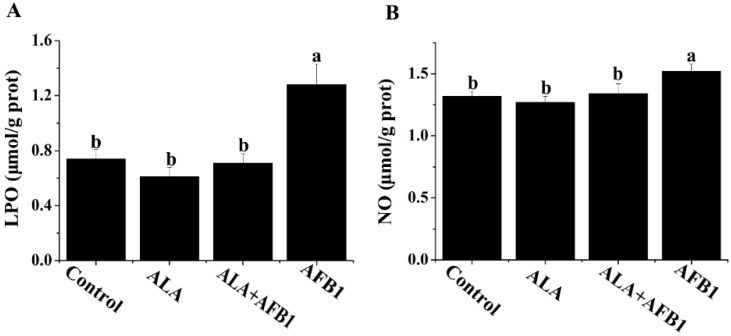
Effect of lipoic acid on oxidative stress (LPO and NO) of liver in broilers fed a diet containing aflatoxin B_1_ (AFB_1_). Values are means ± SE (*n* = 8 per group). Columns with different letters are significantly different (*p* < 0.05). (**A**) LPO, lipid peroxide; (**B**) NO, nitric oxide; SE, standard error.

The results of the biochemical analyses for the liver lipid peroxides (LPO) and nitric oxide (NO) levels are shown in Figure 2. The livers of birds fed AFB_1_-contaminated diets showed a significant increase in LPO and NO (*p* < 0.05, Figure 2). Treatment with α-LA (300 mg/kg) plus AFB_1_ succeeded in inhibiting the elevation of LPO and NO in the liver and made them approach normal levels.

### 2.3. Effects of α-LA on the mRNA Levels of Proinflammatory Genes (TNF-α and IL6) in Livers of Chickens Exposed to AFB_1_

**Figure 3:**
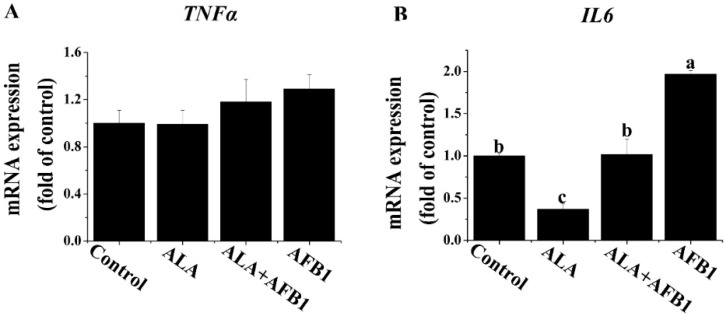
Effect of lipoic acid on the expression of proinflammation genes (*TNF-*α and *IL6*) of liver in broilers fed on a diet containing aflatoxin B_1_ (AFB_1_). Values are means ± SE (*n* = 8 per group). Columns with different letters are significantly different (*p* < 0.05). (**A**) *TNF-*α, tumor necrosis factor alpha; (**B**) *IL6*, interleukin 6; SE, standard error.

The genes modulating hepatocellular inflammation affect the extent of hepatic injury. We first investigated the effect of α-LA on the proinflammatory gene expression of chronic liver damage by measuring the tumor necrosis factor alpha (*TNF-*α) and interleukin-6 (*IL6*) mRNA levels in broiler chickens exposed to AFB_1_. The results showed an increase in the mRNA level of *IL6* (*p* < 0.05, Figure 3B) and no significant changes in the gene expression of *TNF-*α (*p* > 0.05, Figure 3A) in livers derived from chickens exposed to AFB_1_. Importantly, the supplementation of α-LA markedly inhibited this increase in the *IL6*. The expression of *IL6* measured in the chickens treated with α-LA alone was reduced approximately three times (*p* < 0.05) as compared with the chickens in the control group.

### 2.4. Effect of *α*-LA on the Expression of Hepatic NF-*κ*B p65 and iNOS in Chickens Exposed to AFB_1_

**Figure 4:**
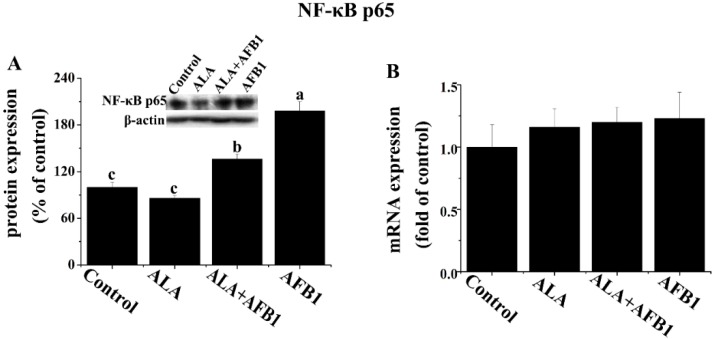
Effect of lipoic acid on the (**A**) protein (*n* = 3 per group) and (**B**) gene (*n* = 8 per group) expression of hepatic transcription factors (NF-κB p65) in broilers fed a diet containing aflatoxin B_1_ (AFB_1_). Values are means ± SE. Columns with different letters are significantly different (*p* < 0.05). NF-κB, nuclear factor kappa B; SE, standard error.

**Figure 5:**
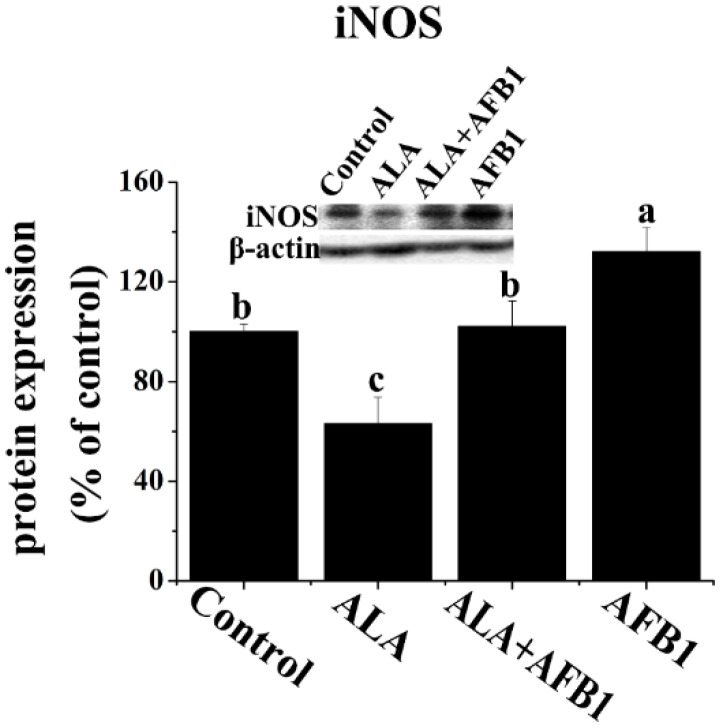
Effect of lipoic acid on the protein expression of hepatic iNOS in broilers fed a diet containing aflatoxin B_1_ (AFB_1_). Values are means ± SE (*n* = 3 per group). Columns with different letters are significantly different (*p* < 0.05). iNOS, inducible nitric oxide synthase; SE, standard error.

In order to determine the effects of α-LA on NF-κB and inducible nitric oxide synthase (iNOS) expression in chickens exposed to AFB_1_, Western blot analysis was carried out in the present study (Figure 4A and Figure 5). AFB_1_ treatment induced an increase in the protein levels of NF-κB *p65* and iNOS (*p* < 0.05, Figure 4A and Figure 5), but had no significant change in the gene expression of *NF-*κ*B p65* (*p* > 0.05, Figure 4B) in the livers derived from chickens. The addition of α-LA into the AFB_1_ diets resulted in a significant suppression of NF-κB and iNOS protein expression (*p* < 0.05, Figure 4A and Figure 5), indicating the anti-inflammatory activity of LA.

## 3. Discussion

Natural toxins probably pose a greater threat to human and animal health than synthetic toxins [15]. Approximately 4.5 billion people worldwide are exposed to AF-contaminated food, particularly in low-income countries. Dietary exposure to AF is among the major hepatocellular carcinoma (HCC) risk factors. Consuming and metabolizing AFB_1_ increases the production of free radicals [16] and lipid peroxidation in the liver [17], which causes liver oxidative damage and inflammation, and results in hepatic damage. α-LA is confirmed to have a number of beneficial effects, preventing and treating some diseases through its action of antioxidant and anti-inflammatory actions [6,13,18]. In our previous work, we found that α-LA had the ability to ameliorate AFB_1_-induced liver injury and oxidative damage, and might contribute to the maintenance of intracellular antioxidants status [19]. However, the precise mechanism(s) by which α-LA attenuates liver injury caused by AFB_1_ has not been completely elucidated. Therefore, the objective of this study was to explore the molecular mechanisms of α-LA protection of the liver from aflatoxicosis. In the present study, the results showed that supplementation of α-LA attenuated hepatic oxidative damage, dramatically inhibited hepatic inflammation, and reduced the protein expression of hepatic NF-κB p65.

Oxidative stress is a risk factor for hepatic injury. The induction of oxidative stress is commonly related to an imbalance between the oxidants and the antioxidant systems. It has been reported that AFB_1_ can initiate the production of free radicals [20], indicating that there is an oxidative pathway involved in the toxicity of AFB_1_. SOD, CAT, and GSH-Px are the crucial antioxidant enzymes responsible for scavenging free radicals in cells. Kanbur *et al.* [21] reported that the administration of aflatoxin significantly decreased the GSH-Px activity in the liver of mice. We recently reported that α-LA enhanced the antioxidant capability of GSH-Px enzymes and elevated the glutathione (GSH) content in the liver of broilers fed AFB_1_-diets [19]. In agreement with this finding, the addition of α-LA into the AFB_1_-diet increased the expression of the *GSH-Px* gene of those birds, compared with that of birds fed the AFB_1_-diet alone. It is noteworthy that GSTα is also one of the most important transferase enzymes involved in AFB_1_-8,9-epoxide detoxification. AFB_1_-8,9-epoxide, an epoxide form of AFB_1_, could bind covalently with cellular macromolecules such as DNA, RNA, and proteins, leading to tissue damage. A decrease in the expression of the hepatic *GSTα* gene, as observed in the chickens exposed to AFB_1_ in the current study, could reduce the ability of liver tissue to conjugate reactive metabolites. The supplementation of α-LA in the AFB_1_-diet tended to up-regulate the expression of the *GSTα* gene (Figure 1E). This enhancement of the expression of the *GSTα* gene may be attributed to an increase in the *de novo* synthesis of GSH, due to the fact that this enzyme can be involved in the synthesis of GSH.

Lipid peroxidation, a common manifestation of oxidative damage, has been observed as a marker of cellular damage due to oxidative stress. In the present study, liver LPO levels increased when birds were exposed to AFB_1_. Supporting our work, it was previously reported that AFB_1_ increased lipid peroxidation in rats [17] and hens [4]. As previously observed by Naaz *et al.* [22], the increase in LPO levels may be attributed to an inhibition of enzymatic antioxidants (e.g., GSH-P_X_ activity) and the depletion of non-enzymatic antioxidants (e.g., GSH) in the livers of the AFB_1_-treated group. Data from the present study suggested that α-LA ameliorated lipid peroxidation, coinciding with a decrease in the thiobarbituric acid reactive substances levels in the serum and livers of rats [23]. These findings suggest that both increased lipid peroxidation and impaired antioxidant system function are closely associated with liver injury, and α-LA protects liver tissue from oxidative damage caused by AFB_1_.

Inflammation is also commonly associated with hepatic injury. A study by Shen *et al.* [16] stated that AFB_1_ could increase the production of ROS such as hydrogen peroxide (H_2_O_2_), hydroxyl radicals (•OH), and superoxide radicals (O^2−^). ROS attacks hepatocytes, leading to the damage of the liver structure and function, which promotes inflammatory response in the liver. Proinflammatory cytokines TNF-α and IL6 play an important role in the process of hepatic inflammation. The induction of an inflammatory reaction (increased production of IL6) is associated with liver injury [24]. In a recent paper published in *Cell*, Park *et al.* [25] demonstrated that IL6 and TNF signaling play a critical role in promoting liver inflammation in dietary and genetic obesity. However, the findings in this study showed that dietary treatments did not change the expression of the *TNF-*α gene, indicating that the *TNF-*α gene may be less sensitive in chickens than that in mammals. This may also be related to the level of AFB_1_ in the diet and the magnitude of liver damage. An increase in the expression of the *IL6* gene was observed in the AFB_1_-treated chickens, indicating that low dosages of AFB_1_ could cause an inflammatory response in the livers of chickens. Supporting this observation, it was previously reported that AFB_1_ increased the production/expression of *IL6* in rats [24] and chickens [26]. In the present study, α-LA alone decreased the mRNA levels of *IL6*, possibly due to its anti-inflammatory characteristics. These results are consistent with those of Zhang *et al.* [27] and Ho *et al.* [18], who have demonstrated various anti-inflammatory effects attributed to α-LA. The mRNA modulation simply suggests a possible modulation of cytokine secretion in this study, but this must be confirmed by looking at the protein levels of cytokines in a further study.

There is increasing concern over the involvement of transcription factors such as the inflammatory transcription factor, NF-κB, in the pathophysiology of various disease processes. It has been suggested that the activation of NF-κB and the induction of NF-κB-dependent gene expression in hepatocytes may contribute to liver damage and inflammatory responses. Therefore, this inflammatory transcription factor may be a possible target of α-LA-mediated protection of hepatoxicity by AFB_1_. It is of interest to mention that NF-κB is activated by oxidative stress, and its activation can be modulated by some antioxidants, possibly through the involvement of the cysteine moiety in p65 of NF-κB [28]. Although α-LA’s inhibitory effects on NF-κB activation, inflammatory response, and oxidative damage are well known [29], this is the first time that α-LA has been reported to have the potential ability to down-regulate AFB_1_-induced NF-κB expression in chicken livers. In the current study, the feeding of the AFB_1_-diet leads to an increase in the protein expression of NF-κB p65 in birds compared with birds fed the control diet. Interestingly, the addition of α-LA into the AFB_1_-diet suppressed the protein expression of NF-κB p65 in the livers of broiler chickens, suggesting that the suppression of NF-κB activation may be responsible for the hepatoprotective effects of α-LA against liver damage induced by AFB_1_. However, we just determined the expression level of NF-κB protein in present study. The effect of LA on the level of NF-κB translocation into the nucleus in AFB_1_ condition should be considered in further study. It is therefore possible that α-LA might be inhibit AFB_1_-induced NF-κB activity, thus reducing the hepatic IL6 production of chickens, which may be related to the suppression of inflammatory responses.

One major consequence of the occurrence of oxidative stress/inflammation is the overproduction of NO that causes tissue damage by reacting with other oxygen radicals. NO is synthesized from l-arginine through the action of iNOS, which is mainly involved in immune response [30]. There is ample evidence that the hepatic overexpression of iNOS plays an important role in liver damage in various liver injury models [20,30]. In addition, mycotoxins other than AFB_1_ are described to modulate iNOS expression such as the trichothecene, deoxynivalenol that affects iNOS expression by the gut, and the brain [31,32]. Our data indicated that AFB_1_ increased the protein expression of iNOS as well as the levels of NO in the livers of broiler chickens. Additionally, α-LA inhibited the increase in the expression of the iNOS protein and lowered the elevation in the production of NO induced by AFB_1_ in the livers. Increased NO production and iNOS activity by aflatoxicosis and the inhibitory effect of α-LA on NO synthesis have previously been reported [8]. The observed decrease in NO levels in the liver may possibly be due to the inhibition of the protein expression of iNOS by α-LA, the expression of which is especially enhanced in tissue damage due to oxidative stress and inflammatory response. Another explanation is that the reduction in NO levels in hepatic cells might be due to the direct scavenging effect of α-LA. In addition, the transcription factor of NF-κB plays a crucial role in the molecular regulation of iNOS expression and in the excessive amounts of NO released [13]. This indicates that the inhibitory effects of α-LA on the release of NO, and the expression of iNOS might be at least partly related to its ability to modulate the NF-κB signaling pathway. However, this does not exclude the possibility that α-LA could regulate the expression of iNOS by other mechanisms, including the stimulation of proinflammatory cytokines. In accordance with these findings, the present study demonstrates that AFB_1_-induced chronic liver injury is alleviated when the NF-κB/iNOS/NO pathway is significantly depressed by α-LA supplementation, which may be linked to both the suppression of inflammatory responses and the prevention of oxidative stress.

## 4. Experimental Section

### 4.1. Collection of Feed Ingredients Contaminated with AFB_1_

A total of 100 feed ingredients sampled from all over the nation were examined for their mycotoxins contents, including AFB_1_, deoxynivalenol, zearalenone, and ochratoxin A using high performance liquid chromatography (HPLC) according to the method of Trucksess *et al.* [33]. Two peanut meal samples, one AFB-free and the other seriously contaminated with AFB_1_ (330 μg/kg), were selected and incorporated proportionally into the basal diet used in this study.

### 4.2. Animals

One-day-old male broiler chickens (Ross 308) were obtained from a commercial hatchery (Chia Tai Co., Ltd, Qinhuangdao, Hebei, China). The brooding temperature and relative humidity were maintained at 35 °C and 65% for the first two days, and then decreased gradually to 21 °C and 45% until 28 days and maintained as such until the end of the experiment. The light regime was 23 L: 1 D. Throughout the rearing period, water and a commercial diet were provided *ad libitum*. The animal care protocol in this experiment followed the commercial management practice and was approved by the Animal Welfare Committee of the China Agricultural University.

### 4.3. Experimental Design

After a 10 day adaption period to the diet and surroundings, a total of 160 eleven-day-old birds with similar body weight (BW) were randomly assigned to four groups with four replicates pens containing ten birds per pen. Four treatment groups included: one group fed the basal diet with 21% normal peanut meal (without any mycotoxins) (control); on group fed a basal diet supplemented with 300 mg/kg DL-α-lipoic acid (Sigma Chemical, St. Louis, MO, USA); on group fed a diet containing 74 μg/kg AFB_1_ (21% moldy peanut meal naturally contaminated with 330 μg/kg AFB_1_ substituting with the same proportion for the normal peanut meal in the basal diet); and one group fed a diet supplemented with 300 mg/kg α-LA and AFB_1_ (determined as 74 μg/kg AFB_1_ without other mycotoxins). All essential nutrients in the basal diet met or were slightly lower than the nutrient requirements recommended by the National Research Council (1994). The composition of diets and the content of AFB_1_ in the diets are shown in Table 1. The feeding trial period lasted for three weeks.

**Table 1:** Composition of the diets during the experiment.

Ingredient	%	Nutrition Component	Content
Corn	57.70	Metabolizable energy (MJ/kg)	12.55
Expanded soybean	6.00	Crude protein (%)	21.50
Soybean meal	8.20	Calcium (%)	0.99
Peanut meal	21.00 ^1^	Total Phosphorus (%)	0.65
Limestone	1.30	Nonphytate Phosphorus (%)	0.43
Dicalcium phosphate	1.80	Methionine (%)	0.62
Salt	0.30	Methionine + Cystine (%)	0.91
Corn oil	2.00	Lysine	1.17
Lysine [98.5%]	0.47	Tryptophan	0.21
DL-Methionine	0.36	Threonine	0.82
Threonine	0.19	Aflatoxin B_1_ (μg/kg)	0/0/74.36/73.44 ^4^
Salinomycin	0.07	-	-
Choline chloride	0.10	-	-
15% Chlortetracycline	0.07	-	-
Mordenzeo	0.11	-	-
Vitamin premix ^2^	0.03	-	-
Mineral premix ^3^	0.30	-	-
Total	100.00	-	-

^1^ AFB-free peanut meal was replaced by AFB-contaminated peanut meal according to the same proportion in trail diets. ^2^ Provided per kilogram of diet: 15,000 IU vitamin A; 3000 IU cholecalciferol; 20 IU vitamin E; 2.18 mg vitamin K_3_; 2.15 mg thiamine; 8.00 mg riboflavin; 4.40 mg pyridoxine; 0.02 mg vitamin B_12_; 25.60 mg calcium pantothenate; 65.80 mg nicotinic acid; 0.96 mg folic acid; 0.20 mg biotin. ^3^ Provided per kilogram of diet: 109.58 mg Fe as ferrous sulfate; 8.14 mg Cu as copper sulfate; 78.04 mg Zn as zinc sulfate; 105.00 mg Mn as manganous oxide; 0.34 mg I as ethylenediamine dihydroiodide; 0.14 mg Se as sodium selenite; 1500 mg choline chloride. ^4^ Four analysis values of AFB_1_ came from the Control, alpha-lipoic acid (ALA), AFB_1_ and ALA + AFB_1_ groups, respectively.

At the end of the experiment, eight chicks with body weights close to the average were selected from each treatment group. The chicks were euthanized humanely by cervical dislocation, and the livers were removed immediately. Liver tissue samples were washed with ice-cold sterilized saline solution, snap frozen in liquid nitrogen and stored at −80 °C for further analysis.

### 4.4. Preparation of Liver Homogenate

Liver tissue (1 g) was cut into small pieces and homogenized in an ice-cold saline buffer (0.85%, pH = 7.4) (1:9, *w*/*v*) with an Ultra-Turrax (T8, IKA-labortechnik, Staufen, Germany) to form homogenates at a concentration of 0.1 g/mL for further analysis. Liver homogenates were centrifuged at 1000× *g* for 15 min at 4 °C, and the supernatants were collected. The supernatants were used for the assays of LPO and NO.

### 4.5. Assay of Oxidant Indices in Liver

The levels of LPO and NO in the liver supernatants prepared as described above and were then measured using colorimetric methods with a spectrophotometer (Biomate 5, Thermo Electron Corporation, Rochester, NY, USA). These levels were determined with the clinical chemistry analyzer (Commercial Kit, Nanjing Jiancheng Bioengineering Institute, Nanjing, China) in accordance with the manufacturer’s recommended procedures. LPO levels were measured by the method of Ohkawa *et al.* [34]. The NO content was determined by a spectroscopic method [35].

The contents of LPO and NO were expressed as μmol per milligram of protein for liver tissue. Total protein content in the liver tissue was determined using a protein assay kit (Jiancheng, Nanjing, China), according to the method of Lowry *et al.* [36] using bovine serum albumin as a standard. The detailed procedure was carried out according to the instructions provided with the detection kit.

### 4.6. Gene Expression Analysis

The mRNA concentrations for broiler chickens including *SOD1*, *SOD2*, *CAT*, *GSH-P_X_*, *GST*α, *HO-1*, *TNF-*α, *IL6*, and *NF-*κ*B p65* were quantified by quantitative real time PCR. β*-actin* was used as a housekeeping gene in this procedure to normalize the gene expression data. The primer information for all the genes is listed in Table 2.

**Table 2:** Gene-specific primer of related genes.

Gene	Genebank Number	Primers Position	Primers Sequences (5′→ 3′)	Product Size
β*-actin*	AW05994	Forward	tgcgtgacatcaaggagaag	300 bp
		Reverse	tgccagggtacattgtggta	
*SOD1*	NM_205064.155	Forward	attaccggcttgtctgatgg	173 bp
		Reverse	cctccctttgcagtcacatt	
*SOD2*	NM_204211.1	Forward	gccacctacgtgaacaacct	208 bp
		Reverse	agtcacgtttgatggcttcc	
*CAT*	NM_001031215.1	Forward	ccacgtggacctcttcttgt	169 bp
		Reverse	aaacactttcgccttgcagt	
*GSH-P_X_*	NM_001163245.1	Forward	cagcaagaaccagacaccaa	156 bp
		Reverse	ccaggttggttcttctccag	
*GST*α	NM_001001776.1	Forward	gagtcaattcggtggctgtt	157 bp
		Reverse	tgctctgcaccatcttcatc	
*HMOX1* *	NM_205344.1	Forward	ggtcccgaatgaatgcccttg	137 bp
		Reverse	accgttctcctggctcttgg	
*TNF*α ^#^	AY765397.1	Forward	tgtgtatgtgcagcaacccgtagt	229 bp
		Reverse	ggcattgcaatttggacagaagt	
*IL6*	NM_204628.1	Forward	agatgtgcaagaagttcacc	286 bp
		Reverse	accacttcatcgggatttat	
*NF-κB p65*	D13719.1	Forward	ttgctgctggagttgatgtc	167 bp
		Reverse	tgctatgtgaagaggcgttg	

* The primer of *HMOX1* was designed according to Druyan *et al*. [37]. ^#^ The primer of *TNFα* was designed according to Hong *et al*. [38].

Total RNAs were extracted from the liver using a TRIZOL Reagent Kit (Invitrogen, San Diego, CA, USA). Reverse transcription was carried out using RT reactions (10 μL) consisting of 500 ng total RNA, 5 mmol/L MgCl_2_, 1 μL RT buffer, 1 mmol/L dNTP, 2.5 U AMV, 0.7 nmol/L oligo d(T), and 10 U ribonuclease inhibitor (TaKaRa, Dalian, China). cDNA was amplified in a 20 μL PCR reaction containing 0.2 μmol/L of each specific primer (Sangon, Shanghai, China) and SYBR green master mix (TaKaRa, Dalian, China). Each cycle consisted of denaturation at 95 °C for 10 s, annealing at 95 °C for 5 s, and extension at 60 °C for 34 s. Each sample was measured in duplicate analysis. If the difference between two duplications was greater than 15%, the sample was analyzed again. The PCR products were verified by electrophoresis on a 0.8% agarose-gel and by DNA sequencing. Standard curves were generated using pooled cDNA from the samples being assayed, and the comparative cycle threshold (CT) method (2^−ΔΔCT^) was used to quantitate mRNA expression as described by Livak and Schmittgen [39].

### 4.7. Western Blotting Analysis

The total protein taken from samples of liver tissue was lysed and homogenized in 500 μL ice radio immunoprecipitation assay buffer (P0013D, Beyotime, Haimen, Jiangsu, China) containing 50 mM Tris (pH 7.4), 150 mM NaCl, 1% NP-40, 0.25% sodium deoxycholate, sodium orthovanadate, sodium fluoride, EDTA, leupeptin supplemented with phosSTOP phosphatase inhibitor (Roche, Basel, Switzerland) and 1 mM Phenylmethanesulfonyl fluoride (ST506, Beyotime, Haimen, Jiangsu, China). Samples were centrifuged at 12,000× *g* for 5 min at 4 °C. Protein concentrations were measured using a BCA Assay Kit (Beyotime, Haimen, Jiangsu, China). After being boiled at 100 °C for 10 min, samples (30 μg protein) containing 1× loading buffer were electrophoresed in a running buffer on a 7.5%–12% SDS Polyacrylamide gel. Separated proteins were then transferred onto polyvinylidene fluoride (PVDF) microporous membrane (Millipore, Billerica, MA, USA) at 80 V for 2 h at 4 °C. After blocking for 1 h in block solution (5% BSA, 0.1% Tween-20 and 0.02% Sodium azide in PBS, pH 7.6) at room temperature, membranes were incubated at 4 °C overnight in primary antibodies against inducible nitric oxide synthase (iNOS) (ab3523) Rabbit polyclonal Ab and NF-κB p65 (C22B4) Rabbit mAb (Cell Signaling Technology, Beverly, MA, USA), and β-actin mouse monoclonal (Beyotime, Haimen, Jiangsu, China). Blots were washed three times and then soaked the membranes with anti-rabbit or anti-mouse IgG-conjugated horseradish peroxidase (Bio-Rad Laboratories, Hercules, CA, USA) at 4 °C for 3 h. After having been washed three times with TBST, immunoprecipitates were detected using SuperSignal West Femto Maximum Sensitivity substrate (Thermo, Pittsburgh, PA, USA). After 5 min, proteins on the membranes were visualized by exposing to X-RAY film (Kodak, Rochester, NY, USA). β-actin was used as an internal control. Western blots were quantified using BioSpectrum 810 with VisionWorksLS 7.1 software (UVP LLC, Upland, CA, USA).

### 4.8. Statistical Analysis

All data were subjected to one-way ANOVA followed by Duncan multiple comparison using a Statistical Analysis Systems (SAS) statistical software package (Version 8e, SAS Institute, Cary, NC, USA). Means were considered significantly different at *p* < 0.05.

## 5. Conclusions

In summary, the findings of this study indicated that liver damage, oxidative stress, and the inflammatory responses were caused by consuming diets containing AFB_1_ at concentrations as low as 74 μg/kg. Additionally, we demonstrated that α-LA protected the liver from AFB_1_-caused injury *in vivo* by attenuating hepatic oxidative damage, suppressing hepatic inflammatory response, and inhibiting the NF-κB p65 expression in broiler chickens. These results may provide a novel insight into the mechanisms of α-LA regarding its ability to protect the liver, indicating the possible potential application of α-LA as a feed additive, in ameliorating the *in vivo* detrimental effects of AFB_1_ toxicity in poultry. Moreover, these findings may further benefit the α-LA related drug development for the future prevention and treatment of aflatoxicosis in humans and animals.
